# Helitrons are enriched in lichenized fungi with long generation lengths and small distribution sizes

**DOI:** 10.1093/g3journal/jkag153

**Published:** 2026-06-16

**Authors:** Julianna Paulsen, Stephen T Sharrett, Devin Mumey, Elaine M Larsen, Nguyen Khoi Nguyen, James Lendemer, Lalita M Calabria, Jordan R Hoffman, Krisztian Magori, Jessica L Allen

**Affiliations:** Department of Biology, Eastern Washington University, Cheney, WA 99004, United States; Department of Biology, Eastern Washington University, Cheney, WA 99004, United States; Department of Environmental Studies, The Evergreen State College, 2700 Evergreen Parkway NW, Olympia, WA 98505, United States; Department of Biology, Eastern Washington University, Cheney, WA 99004, United States; Department of Biology, Eastern Washington University, Cheney, WA 99004, United States; Department of Biology, Eastern Washington University, Cheney, WA 99004, United States; Department of Botany, Research & Collections, The New York State Museum, Albany, NY 12230, United States; Department of Environmental Studies, The Evergreen State College, 2700 Evergreen Parkway NW, Olympia, WA 98505, United States; Division of Science and Mathematics, Delta College, University Center, MI 48710, United States; Department of Biology, Eastern Washington University, Cheney, WA 99004, United States; Department of Biology, Eastern Washington University, Cheney, WA 99004, United States; Conservation and Research Department, Santa Barbara Botanic Garden, Santa Barbara, CA 93105, United States

**Keywords:** conservation genomics, comparative genomics, symbiosis

## Abstract

Transposable elements have the potential to drive genome evolution by introducing mutations and causing structural instability and chromosomal rearrangements, particularly under conditions like environmental or genetic stress. In this study, we generated 18 new long-read-based metagenomically assembled reference genomes for lichenized fungi, which form obligate mutualistic symbioses with algae or cyanobacteria. We used the new genomes and 10 publicly available genomes to investigate the relationships between species traits (i.e. dominant reproductive mode, distribution size, and generation length) and the abundance and spatial distribution of transposable elements using a phylogenetic comparative framework. We found that species with smaller distribution sizes and longer generation lengths had a higher genomic DNA transposon load. Specifically, their genomes were enriched with Rolling Circle transposons, which contradict previous research that has identified high proportions of retrotransposons in rare species. Disproportionate distributions of transposable elements in rare and range-restricted species may disrupt genomic stability, decrease fitness, and be reflective of species experiencing a greater degree of stress. Conversely, greater transposable element activity may be an important source of novel genetic diversity in isolated populations with limited gene flow. Further research is needed to understand the potential mechanisms driving transposable element proliferation in rare species’ genomes and if transposable element content is predictive of increased extinction risk.

## Introduction

Transposable elements (TEs) are genes that encode their own machinery to move within a genome (Wells and Feschotte 2020). TEs are a type of eukaryotic mobile genetic element that are divided into 2 major classes. Class I TEs, called retroelements or retrotransposons (RTs), are distinguished by the use of an mRNA intermediate to complete transposition, and are often described as “copy and paste” elements. Class II elements, called DNA transposons (DTs), do not use an mRNA intermediate, and instead transpose directly through a mechanism described as “cut-and-paste.” DTs are generally not self-replicative, but are capable of utilizing host machinery to increase their copy number in a genome. One mechanism by which DT proliferation occurs is during synthesis in interphase, where DTs can move from replicated to unreplicated chromosomes, duplicating themselves in the process (Zaratiegui 2017). TEs are subclassified by the specifics of their genetic structure, such as variations in the genes and proteins involved in these processes, insertion site preferences, and the presence of accessory genes (Wicker et al. 2007). Helitrons, for example, are a type of DT that transpose via a rolling-circle mechanism using the RepHel protein, enabling them to self-replicate during each transposition event (Barro-Trastoy and Köhler 2024). Subclass characteristics affect the transposition efficiency, replication, and selective pressures related to host fitness (Bourgeois et al. 2020; Duhamel et al. 2023).

TE activity has the potential to facilitate genomic restructuring and evolution by reorganizing genes and chromosomes and causing nucleotide mutations (Aksenova et al. 2013; de Jonge et al. 2013; Kent et al. 2017; Wong et al. 2019; Rahnama et al. 2020; Almojil et al. 2021). The relationship between host defenses that prevent this genomic remodeling and TE evasion is often described as an evolutionary arms race, where both parties engage in a rapid diversification of strategies to outpace the other (Koonin et al. 2020). One such strategy employed by eukaryotic hosts to curb the potentially deleterious effects of TE transposition, RNA interference (RNAi), includes silencing of TEs by specialized RNA molecules, which recruit DNA methylation machinery or bind to transcripts directly and trigger cleavage (Lax et al. 2020). In fungi, specific pathways of RNAi-mediated TE deactivation include cotranscriptional RNA surveillance processes, somatic quelling, and meiotic silencing (Gladyshev 2017). Repeat-induced point mutation (RIP) and methylation-induced premeiotically are 2 fungi specific TE deactivation pathways that both utilize cytosine methyltransferases to induce methylation and, in the case of RIP, induction of C to T mutations, to deactivate TEs (Gladyshev 2017; van Wyk et al. 2021; Romeijn et al. 2026). TE success in eukaryotes is intrinsically tied to host fitness, as TEs must proliferate in the germline to persist within a population. Thus, TEs self-regulate their expression using encoded transcriptional regulators and modify their insertion preferences (Bonnet and Lesage 2021; Hermant and Torres-Padilla 2021). Stress is one factor that is known to modulate host-TE dynamics, and it is believed that cellular stress responses can divert resources away from quelling TEs, allowing their proliferation in the genome (Fouché et al. 2019; Mustafin and Khusnutdinova 2019).

Studies of single species have reported high genomic TE loads in rare and endemic organisms across kingdoms (Ruiz-Ruano et al. 2021; Linscott et al. 2022; Wang et al. 2023), but what drives this pattern is not known. Environmental conditions and population structure are traits correlated with rarity that may be connected to TE abundance in the genome (Abascal et al. 2016; Ruiz-Ruano et al. 2021). Additionally, certain classes of TEs, such as RTs, may be correlated with adverse environmental conditions to which rare species are often subjected (Cavrak et al. 2014; Milyaeva et al. 2023). Using multispecies comparative frameworks will facilitate a better understanding of the relationship between rarity and TEs, which is pertinent for conservation efforts, as large-scale alterations of the genome facilitated by TEs can decrease fitness and increase the rate of speciation (Ricci et al. 2018; Serrato-Capuchina and Matute 2018). In the case of endangered organisms, either outcome translates to increased extinction risk.

Lichens are obligate mutualistic symbioses composed of dominant fungal, algal, and/or cyanobacterial symbionts (Brodo et al. 2001). Recent research has shown that lichens consist of a variety of associated microbes and macroscopic organisms in addition to the main fungal and photosynthetic partners (Grimm et al. 2021; Allen and Lendemer 2022). Lichens exhibit diverse life history traits that underlie their interactions with the environment, such as different reproductive strategies, substrate preferences, growth forms, and symbiotic associations (Manzitto-Tripp et al. 2022). Due to their relevance as quintessential symbioses and their ability to produce diverse and biotechnologically relevant secondary metabolites, lichen genomics has become a popular avenue of research (Allen and Lendemer 2022; Singh et al. 2025). However, their TE landscapes remain less well characterized than other taxonomic groups. In the small number of published, long-read genomes of lichenized fungi where TE content has been reported, RTs are identified as the most abundant TE, and total TE content ranges from 8% to 33% (Mckenzie et al. 2020; Allen et al. 2021; Heuberger et al. 2024). This is in line with fungal genomes more broadly, which are reported to range from 0% to 45%, the majority of which are also identified as RTs (Castanera et al. 2017).

In this study, we investigate the relationship between TEs and rarity, reproductive mode, generation length, and distribution size in lichenized fungi using a multispecies, comparative genomics framework. Assigning a species rarity requires assessing multiple, nuanced factors (Crisfield et al. 2024). Thus, while one predictor in this study was the assignment of “rare” or “common” to each species, we used additional, more narrowly circumscribed traits that are often associated with rarity (i.e. reproduction, generation length, and distribution size) as additional predictors. Using genomes generated via long read sequencing for 26 different species of North American Lecanoromycetes lichenized fungi, 18 of which represent previously unpublished sequences, we identified TEs, calculated multiple metrics for rarity, and divided them into groups based on rarity-associated life history traits. We hypothesized that TEs, specifically RTs, would be present in greater abundance in the genomes of rare species. We expect that rare species experience greater degrees of environmental and genetic stress than common species, resulting in unchecked self-replication of TEs. We tested this hypothesis by comparing the number of TEs and the percent of the genome occupied by TEs for each species in a phylogenetically informed statistical framework. Additionally, we compared the spatial distribution of TEs, hypothesizing that rare species would experience higher instances of colocalization of genes and TEs. To better understand intraspecific variation in the species with the highest TE content, we compared the TE abundance and genomic structural variation between 2 *Pseudocyphellaria rainierensis* individuals from different populations. We found that DT abundance was significantly associated with distribution size and generation length. We also found that Helitrons were enriched in the genomes of species with small distribution sizes and long generation lengths.

## Methods

### Genome generation and annotation

We generated 18 new metagenomically assembled genomes (hereafter “genomes”) in this study (Table 1). Genomes were sequenced and analyzed using standard lab procedures published in Pfeffer et al. (2023). After collection, specimens were air dried, then either frozen until extraction or extractions were initiated within 24 h of collection. A single lichen thallus was divided into 16 tissue disruption tubes, and DNA extraction was performed using the DNeasy Plant Pro Kit (Qiagen, Germany) according to the manufacturer's protocol with minor modifications. Separation into multiple tubes is required to yield sufficiently high molecular weight DNA for long-read sequencing. TissueLyser II (Qiagen, Germany) was used to disrupt dry material, and this step was repeated until the specimen was thoroughly homogenized. Resultant DNA was eluted using 50 μL of elution buffer, and the final extracts were pooled into a single tube per species. This process was repeated for all 18 new genomes. Any remaining tissue after DNA extraction was deposited in the Eastern Washington University herbarium. DNA was quantified with the Qubit Fluorometer 4.0 using the 2× HSdsDNA reagents following the manufacturer's protocol (Thermo Fisher Scientific, United States). To select for high-molecular-weight DNA fragments, a 0.4:1 volumetric ratio of Mag-Bind TotalPure NGS magnetic beads (Omega BioTek, United States) to elute was used for size selection in all specimens except *Sticta beauvoisii*, where a 0.8:1 ratio was used due to initially low DNA yield. Sequencing was completed using the Nanopore MinION Mk1B (Nanopore, United Kingdom). Oxford Nanopore Technologies Ligation sequencing kit (LSK-114) library preparations were completed according to the manufacturer's protocol for the majority of genomes (Nanopore, United Kingdom). Genome sequencing for *Lepraria finkii*, *Lepraria lanata*, *Lepraria normandinoides*, *Lepraria oxybapha*, *Punctelia appalachensis*, *Punctelia rudecta*, *Usnea strigosa*, and *Usnea subfusca* were completed by Cold Spring Harbor Laboratories on the Oxford Nanopore Technologies PromethION platform. Basecalling was completed with Dorado v0.9.6 (Nanopore, United Kingdom), and assembly of metagenomic data was completed using Flye v2.9.5 in meta mode (Kolmogorov et al. 2020). Metagenomes were filtered based on the workflow described in McKenzie et al. (2020), which relies on identifying the dominant fungal symbiont through sorting contigs based on depth, GC content, and homology searches. Genome completeness was assessed using BUSCO v6.0 (Manni et al. 2021), with the ascomycota_odb12 database (Tegenfeldt et al. 2024). Genomes were annotated for repetitive regions using RepeatModeler2 v2.0.6 (Flynn et al. 2020) and Repeatmasker v4.1.8 (Smit et al. 2013). All other annotations were conducted using Funannotate v1.8.7 for cleaning, masking, and predicting, and v1.8.17 for final annotations (Palmer and Stajich 2020). Biosynthetic gene clusters (BGCs) were annotated with AntiSMASH v6.1.1 separately from the funannotate pipeline (Blin et al. 2025). Annotations for repetitive regions, BGCs, and remaining genes were merged into a single annotation file with the Funannotate decorate.gff3 function. Note that the Funannotate clean function did not identify any repetitive contigs in any genomes, and thus simply renumbered contigs from largest to smallest. To ensure all contig names were correctly synchronized across assemblies used in each annotation program, we used Minimap2 v2.28 (Li 2018) to align contigs and identify any name mismatches. If needed, contig names were then reassigned in gff3 to align with the names in the output of Funannotate clean using a custom bash script.

**Table 1.** Genome metrics for newly generated genomes, previously published internal genomes, and external genomes acquired from GenBank.

	Total genome size	Number of contigs	N50	GC content	Number of genes	Number of tRNAs	Complete BUSCOs	Single BUSCOs	Duplicate BUSCOs
*Cladonia chlorophaea*	42,625,121	523	148,102	45.29	12,952	99	97.3	75.4	21.9
*Cladonia imbricarica*	36,666,740	37	1,603,632	42.92	8,954	60	98.5	98.3	0.2
*Cladonia rangiferina*	38,484,624	36	1,569,438	42.98	9,923	57	98.4	98.3	0.1
*Diploschistes muscorum*	51,546,914	111	1,537,671	41.23	8,401	108	96.4	95.3	1.2
*Lepraria finkii*	40,852,740	32	4,154,807	46.58	9,409	84	98.2	97.9	0.2
*Lepraria lanata*	47,102,205	42	2,355,529	45.6	9,775	62	98.8	98.6	0.1
*Lepraria normandinoides*	29,300,421	162	403,430	48.41	7,927	137	96.5	94.4	2.1
*Lepraria oxybapha*	43,363,478	109	1,210,317	46.18	9,144	82	98.7	97.9	0.8
*Pseudocyphellaria citrina*	51,635,776	44	2,349,121	47.17	13,698	84	98	96.5	1.5
*Pseudocyphellaria rainierensis (Surp. Creek)*	125,582,678	26	7,148,722	39.02	13,824	64	95.6	94.3	1.3
*Pseudocyphellaria rainierensis (Col. Bob)*	131,031,989	19	7,147,418	39.13	14,480	46	98.3	96.8	1.5
*Punctelia appalachensis*	45,689,027	25	2,208,748	40.97	9,991	65	97.8	97.6	0.2
*Punctelia rudecta*	42,749,237	31	1,874,871	43.77	11,857	58	98	97.9	0.1
*Sticta beauvoisii*	46,674,901	184	677,788	47.71	10,458	57	97.3	96.9	0.5
*Sticta deyana*	43,509,696	127	891,764	48.13	10,601	60	96.3	95.3	1
*Sulcaria isidiifera*	71,390,556	23	3,455,340	32.4	10,429	91	98.6	98.5	0.1
*Usnea strigosa*	43,172,064	32	2,168,579	42.49	10,511	89	94	93.7	0.2
*Usnea subfusca*	49,089,431	30	2,173,746	41.37	10,855	64	98	97.8	0.2
Previously published genomes (internal)									
* Bacidia gigantensis*	33,117,897	24	1,807,239	44.67	8,942	51	97.3	97.2	0.1
* Lepraria neglecta*	42,023,604	11	5,201,525	47.39	11,669	43	98.3	97.9	0.4
* Letharia columbiana*	5.23E + 07	161	666,803	39.57	11,564	76	97.6	94.2	3.4
* Letharia lupina*	49,242,005	31	2,098,233	38.73	10,214	60	98.1	97.8	0.3
Externally published genomes									
* Acarospora socialis*	31,172,923	38	2,402,664	47.3	8,587	109	98.9	98.6	0.3
* Bacidia rubella*	33,524,925	51	1,771,855	45.28	9,589	45	97.1	96.9	0.3
* Cladonia perforata*	33,606,327	72	1,102,137	47.06	8,624	46	98.2	98.1	0.1
* Niebla homalea*	50,566,743	52	1,266,640	37.96	9,409	67	96.7	96.5	0.2
* Ramalina farinacea*	32,742,913	44	1,546,935	46.69	9,087	64	96.7	96.5	0.2
* Umbilicaria muhlenbergii*	34,812,353	7	7,009,248	47.12	8,148	35	98.1	97.8	0.2

### Metadata collection

Trait data, including rarity (rare or common), reproductive mode (sexual or asexual), generation length (short, medium, or long), and distribution size (m^2^), were compiled from expert knowledge, publications, and federal data (Table 2). Rarity was assigned by holistically assessing various life history traits, including those analyzed in this study, as well as additional factors like patchiness, abundance, and changes in population sizes that can be evaluated by field observations but are not easily collapsible into a discrete categorical or numerical trait. Therefore, rarity as it is used here captures more ecological nuance than any one single trait alone, allowing us to evaluate whether specific traits or combinations thereof are more influential on TE constituency. Rarity-related traits (here, reproductive mode, generation length, and distribution size) were also used as predictive variables in our models to identify more specific trends and avenues for future hypotheses about the relationship between TEs and life history traits in lichens. Generation length was defined based on methods from Yahr et al. (2024), where species are grouped into categories of generation length based on their life history strategies. Distribution sizes were calculated in ArcGIS v3.30 (ESRI 2024) by georeferencing expert-drawn distribution maps or referencing published species descriptions (Table 2), transforming them into shapefiles, clipping the polygons to the North American continental landmass, and erasing all terrestrial water features using shapefiles from the Commission for Environmental Cooperation (Montreal, CA; 2022 and 2023). To improve the accuracy of area calculation, geospatial data was projected onto an Albers equal area conic basemap. The final area of each polygon was reported in square meters using the calculate geometry tool, and this data was exported using a custom Python script.

**Table 2.** Species metadata including collection info for new genomes, source publication for previously published genomes, trait data used in this study, and sources for distribution sizes and trait data for each species.

Species name	Collection metadata	Distribution size (km^2^)	Generation length	Reproductive mode	Rarity	Trait data sources
*Cladonia chlorophaea*	*Pinus ponderosa savanna*, *Palisades park*, *Rimrock Trail*, *Washington*, *Spokane Co. (47.656N,* −*117.484W; Devin Mumey 420)*	13,725,112.52	Short	A	C	Tripp and Lendemer (2020)
*Cladonia imbricarica*	*Pinus ponderosa savanna, Dishman Hills Conservation Area, just west of Iller Creek Trailhead, Washington, Spokane Co. (47.602N,* −*117.283W; Devin Mumey 407-A)*	1,035,578.519	Short	A	R	Brodo et al. (2001)
*Cladonia rangiferina*	Exposed soil outcrop embankment along infrequently traveled roadside. Washington, Chelan Co., Leavenworth, Wenatchee Nat Forest, Roadside of NF-6701 past intersection with NF-6700, between the bridge over Rainy Creek and other river further along the road (47.8595N, −120.9835W; Jordan Hoffman s.n.)	12,778,158.25	Short	A	C	Tripp and Lendemer (2020)
*Diploschistes muscorum*	Turnbull Lab for Ecological Studies, 0.12 mi NE of turnoff of S. Badger Lake Road, 1.5 mi S of intersection between Lt. Col. Michael P. Anderson Memorial Highway and Cheney Plaza Road. Washington, Spokane Co., Cheney (47.45481N, −117.5744W; Jessica Allen 5013)	14,407,856.16	Long	S	C	Tripp and Lendemer (2020)
*Lepraria finkii*	Great Smoky Mountains National Park, Lumber Ridge Trail ∼2.3 mi NE of Tremont Institute, S slopes of Lumber Ridge above Bloody Branch of Meigs Creek. Mixed hardwood (*Acer saccharum, Carya, Oxydendrum, Quercus alba, Q. rubra*)—conifer (*Pinus*, *Tsuga*) forest with *Kalmia-Rhododendron* understory. On *Quercus rubra* base (35.6531N, −83.6711W; James Lendemer 71,953)	11,214,498.23	Short	A	C	Tripp and Lendemer (2020)
*Lepraria lanata*	NW-facing overhang on Cattail Peak, North Carolina (35.7964N, −82.2569W; Allen s.n.)	28,112.47664	Medium	A	R	Tripp and Lendemer (2020)
*Lepraria normandinoides*	Vertical shale outcrop at the pigeon River Whitewater Boat Launch, Tennessee (35.7756N, −83.101W; Hollinger 27,155)	1,361,504.869	Short	A	C	Tripp and Lendemer (2020)
*Lepraria oxybapha*	Quartzite outcrop in Great Smoky National Park, on a roadbank off Old Cataloochee Turnpike, North Carolina (35.6465N, −83.0633 W; Hollinger 27,129)	1,369,424.654	Short	A	C	Tripp and Lendemer (2020)
*Pseudocyphellaria citrina*	United States of America, Washington State, Alpine Lakes Wilderness, Taylor River Trail, FS trail 1002 (47.57408N, −121.42597W; Gifford (Marco) Pinochet IV s.n.)	787,956.7691	Long	A	C	Lücking et al. (2017)
*Pseudocyphellaria rainierensis (Col. Bob)*	*Tsuga heterophylla-Abies amabilis/Polystichum munitum-Vaccinium ovalifolium stand with varying levels of riparian influence. Moderate to dense coarse woody debris. Understory sparse/patchy with lots of Rubus spectabilis along riparian areas. Colonel Bob Trail #851, near intersection with Ziegler Creek, 3 mi from TH, Colonel Bob Wilderness, Olympic National Forest, Washington (47.47786N,* −*123.763650W; Stephen Sharrett 105)*	259,693.6853	Long	A	R	Lücking et al. (2017)
*Pseudocyphellaria rainierensis (Surp. Creek)*	*Tsuga-Thuja plicata climax stand with scattered Abies amabilis and ripiarian influence. Moderate to dense coarse woody debris. Lots of recent windfall. Open understory—dominanted by Vaccinium ovalifolium/Cornus canadensis/Clintonia uniflora. Lots of small seeps/stream running south lined with Rubus spectabilis/Oplopanax horridus. Surprise Creek. NE of the Surprise Creek Trail #1060. Approx. 0.42 mi S of the Surprise Creek Trail TH, 4.51 mi SSW of Steven's Pass. Alpine Lakes Wilderness, Washington (47.7018N,* −*121.15705W; Stephen Sharrett 903)*	259,693.6853	Long	A	R	Lücking et al. (2017)
*Punctelia appalachensis*	Cataloochee divide, Great Smoky Mountain National Park, North Carolina (35.585N, −83.074W; Allen 6097)	187,104.6771	Medium	A	R	Tripp and Lendemer (2020)
*Punctelia rudecta*	Cataloochee divide, Great Smoky Mountain National Park, North Carolina (35.585N, −83.074W; Allen 6098)	3,587,472.473	Medium	A	C	Tripp and Lendemer (2020)
*Sticta beauvoisii*	Metasandstone outcrop, Locust Run Creek, Virginia (38.7240N, −77.9273W; Harris s.n.)	544,060.7335	Medium	A	C	Brodo et al. (2001)
*Sticta deyana*	Sipsey wilderness area, Alabama (34.3241N, −87.4513W; Tripp 6599)	1,552.481037	Long	A	R	Tripp and Lendemer (2020)
*Sulcaria isidiifera*	Elfin Forest, California (35.3325N, 120.8238W; Balderas 70)	36.19660427	Long	A	R	McMullin et al. (2019)
*Usnea strigosa*	Cataloochee divide, Great Smoky Mountain National Park, North Carolina (35.582012, N, 1,483.07037; Allen 6099)	2,003,165.168	Medium	S	C	Tripp and Lendemer (2020)
*Usnea subfusca*	West slopes of Jack Mountain, Highlands Wildlife Management Area, Virginia (38.3746, −79.5034; Allen 6100)	48,785.13062	Medium	S	R	Tripp and Lendemer (2020)
**Previously published genomes**						
**Species name**	**Genome source citation**					**Sources**
*Bacidia gigantensis*	Allen et al. (2021)	209.1420958	Medium	S	R	Allen et al. (2021)
*Lepraria neglecta*	Pfeffer et al. (2023)	10,426,807.23	Short	A	C	Tripp and Lendemer (2020)
*Letharia columbiana*	McKenzie et al. (2020)	1,202,898.439	Medium	S	C	Altermann et al. (2016)
*Letharia lupina*	McKenzie et al. (2020)	2,395,797.465	Medium	A	C	Altermann et al. (2016)
*Acarospora socialis*	Adams et al. (2023)	6,249,824.495	Long	S	C	Nash et al. (2002)
*Bacidia rubella*	Gerasimova et al. (2022)	1,152,705.446	Medium	S	C	Tripp and Lendemer (2020)
*Cladonia perforata*	Leavitt et al. (2023)	12,123.31399	Short	A	R	Tripp and Lendemer (2020)
*Niebla homalea*	Duong et al. (2021)	43,307.77313	Long	S	R	Brodo et al. (2001)
*Ramalina farinacea*	Llewellyn et al. (2023)	1,414,099.471	Medium	A	C	Brodo et al. (2001)
*Umbilicaria muhlenbergii*	Park et al. (2014)	4,686,307.09	Long	S	C	Tripp and Lendemer (2020)

### Comparative genomics analysis

Comparative genomics analyses were performed using 18 newly generated genomes, four genomes previously published from our research group (McKenzie et al. 2020; Allen et al. 2021; Pfeffer et al. 2023), and six publicly available genomes acquired from GenBank (Table 1), totaling 28 genomes. Two genomes of the species *P. rainierensis* were generated for individuals from different populations (Colonel Bob Wilderness, Washington, USA (Col. Bob); Surprise Creek, Washington, USA (Surp. Creek)) to investigate intraspecific variation in genomic TE content. One genome, *Cladonia chlorophaea*, was not sufficiently complete and was thus not included in any statistical analyses.

The final dataset for TE analysis consisted of 27 genomes representing 26 different species. All previously published genomes were reannotated according to the methods above to ensure standardized comparisons across genomes. We first sought to build a robust phylogeny to place all of our analyses in a comparative phylogenetic framework. To build the phylogeny, we used a total of 50 genomes of Lecanoromycetes lichenized fungi and closely related ascomycetes, utilizing *Saccharomyces cerevisiae* and *Schizosaccharomyces pombe* as outgroups. This included the 28 genomes in our dataset, 21 additional genomes generated with short-read data originating from Gerasimova et al. (2022), and the long-read reference genome for *Pseudevernia furfuracea* (GCA_022315425.1) acquired from GenBank. Orthofinder v2.5.5 was then used to infer phylogenetic relationships using orthologous genes (Emms and Kelly 2019). Phytools v2.4.4 was used to load and configure the species tree in R for subsequent analyses (Revell 2011). Because contiguous repetitive sequences cannot be as accurately assembled with short-read data or be spatially localized in reference to genes across the genome, we trimmed the species tree to only include long-read reference genomes for subsequent analyses.

Because related species tend to be more similar in terms of genetic traits than unrelated ones, we tested for phylogenetic signal in our dataset prior to conducting statistical tests using the R package phyr v1.1.0 using the pglmm_compare() tool without a predictor variable using a binomial distribution and a Poisson distribution for percent of genome and number of TEs, respectively (Li et al. 2020). All resulting test statistics are reported in Supplementary Table 1. In short, there was significant phylogenetic signal detected in the percent of occupancy of DTs and in counts of total TEs and RTs, while there was no significant phylogenetic signal in the remaining datasets, suggesting that shared ancestry contributed little to the results. The presence of statistically significant phylogenetic signal in the models justified the use of phylogenetically informed statistical tests for downstream analyses (Supplementary Table 1). We tested for correlations among trait data using nonparametric statistical methods: Chi-square tests for associations between 2-level categorical traits, Wilcoxon rank-sum tests for comparisons between continuous variables and 2-level categorical traits, and Kruskal–Wallis tests for comparisons involving continuous variables and 3-level categorical traits. Our analyses revealed a significant relationship between only rarity and log-transformed distribution size (Supplementary Table 2). Univariate phylogenetic generalized linear mixed models (PGLMM) from the package phyr v1.1.0 (Li et al. 2020) were used to test the relationships between both continuous and discrete trait data and both metrics of TE constituency, number of elements, and percent of genome occupied. When the number of elements was the response variable, a Poisson PGLMM was used. For tests where the percentage of the genome occupied was the response, percentages were converted to successes and failures so that a binomial PGLMM could be used. We included both metrics of TE constituency because the number of elements is only informative regarding the abundance of TEs in each genome, while the percentage of the genome occupied has the additional nuance of the length of the TE insertions themselves. To assess if RTs are enriched in the genomes of rare species in relation to DTs, we calculated the percentage of total TEs that were RTs for each species and conducted binomial PGLMM analyses on the resulting proportions. Distribution size was log-transformed for all analyses due to the presence of extremely low and high values. We corrected for increased family-wise type I error using a Bonferroni correction within each set of analyses.

### TE spatial distribution analysis

**Fig. 1.**
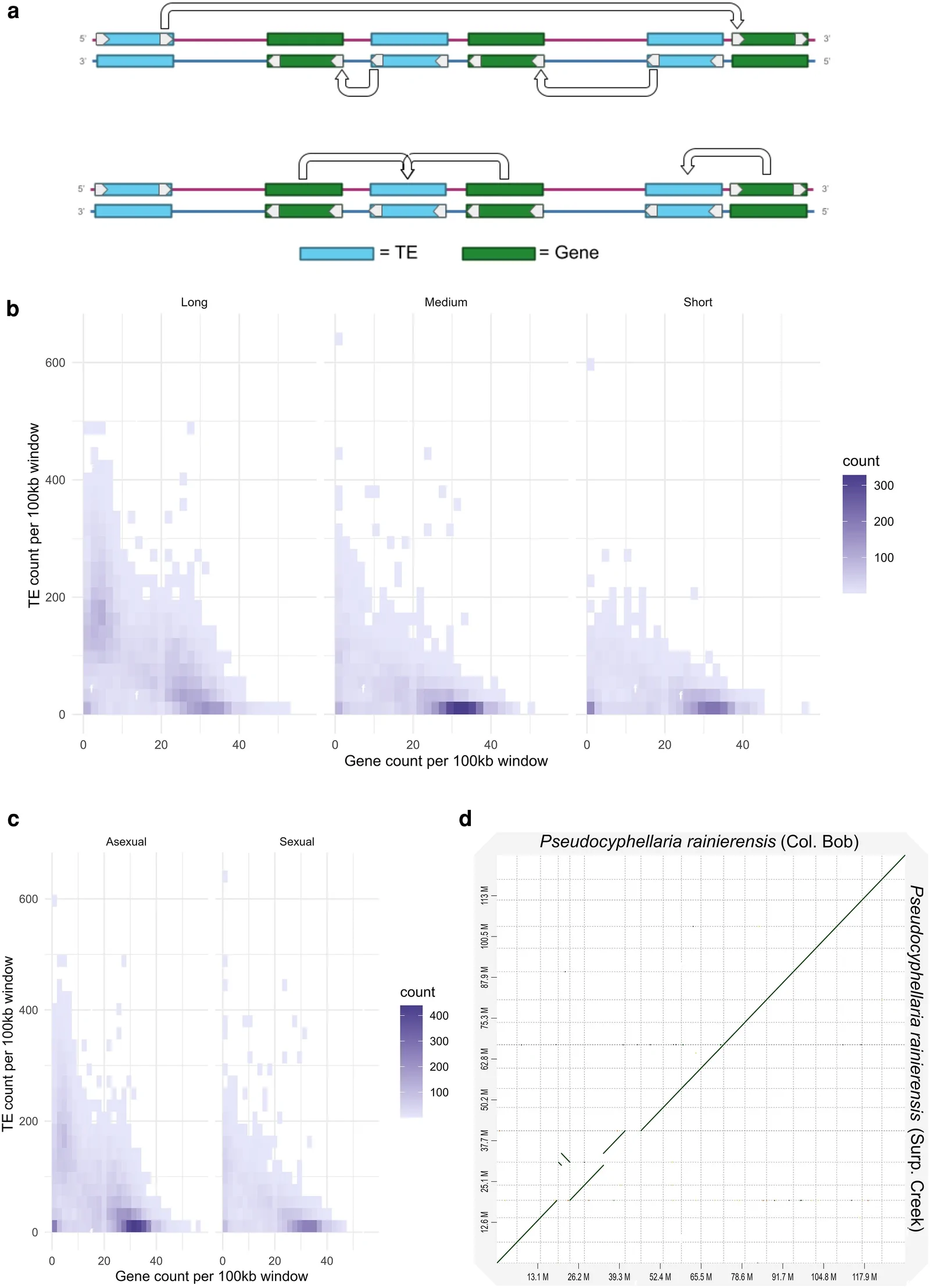
Transposable element impacts on genome structure. a) Schematic representation of the method to calculate mobile genetic element (TE) gene colocalization. The first analysis (top) was conducted by calculating the distance between the end of each gene (blue) and the start of the nearest TE (green) on the same strand, in the direction of transcription, and within the same contig. The second analysis was conducted by calculating the distance between the midpoint of each TE and the nearest gene, regardless of strand and direction. b) Heatmap of the number of 100 kb windows in each genome (represented by the gradient of low values (light purple) to high values (dark purple)) with a specific number of genes (*x* axis) and TEs (*y* axis) by generation length. If differences in TE gene colocalization were observed, it would be expected that high values of the color gradient would be present in different quadrants of the graphs for each group. c) The same gene-TE distance heatmap described in (b) faceted by reproductive mode. d) Dotplot of the alignment of 2 *Pseudocyphellaria rainierensis* genomes collected and sequenced from 2 different populations. Gridlines delineate contigs. Total genome length is represented on the bottom and left axes.

To determine if genes and TEs tend to colocalize more often in rare species than common ones, we analyzed the spatial distribution of TEs relative to genes. General Feature Format (GFF3) files containing the annotations generated by Funannotate (Palmer and Stajich 2020) and RepeatModeler2 (Flynn et al. 2020) were loaded into R as GRanges objects using the R package GenomicRanges v1.54.1 (Lawrence et al. 2013) and ape v5.8 (Paradis and Schliep 2018). We calculated the distance between each TE and the nearest gene on the same contig, and summarized the mean of this value for each species. This analysis was then repeated after subsetting the data by strand, such that the distances were calculated between TEs and the nearest gene on the same strand and in the direction of transcription to capture TE association with both primary and quaternary genomic structures, where transcription levels can vary (Fig. 1a). Mean distances were corrected for differences in genome size and gene density by multiplying the values by the total number of genes by total base pairs per genome. To assess the relationship between average TE to gene distances and trait data, we used a univariate Gaussian PGLMM.

To take an in-depth look at the role of TEs in intraspecific variation, we aligned the genomes of 2 individuals of *P. rainierensis* collected from different populations and assessed the resulting alignment for the presence of structural variants (SVs) caused by TEs. To serve as a comparison, the same analysis was conducted on one closely related species pair in the dataset, *S. beauvoisii* and *Sticta deyana* (Supplementary Fig. 1). Minimap2 v2.28 (Li 2018) was used to create the alignment, Sniffles2 v2.6.0 (Smolka et al. 2024) was used to identify and visualize SVs, and D-Genies v1.5.0 (Cabanettes and Klopp 2018) was used to visualize the alignments.

## Results

### Genome metrics

Eighteen genomes were newly generated as part of this study, resulting in a total of 1.2 Gb of data after assembly and filtering (Table 1). The average N50 value was 2.4 Mb. The highest N50 was 7.15 Mb for *P. rainierensis* (Surp. Creek), and the lowest value was 148.1 Kb belonging to *C. chlorophaea*. Genome assembly completeness assessments using BUSCO recovered a high number of conserved genes in all genomes (>90%) except *C. chlorophaea*, which was dropped from downstream analyses because the low BUSCO score indicated that the assembly was incomplete and may therefore underrepresent TE constituency. We added 10 published genomes generated with long-read sequence data to our dataset. Thus, our final dataset for the TE analysis contained 27 genomes from 26 different species of lichenized fungi. The complete set of 27 genomes was annotated with the same exact pipeline to ensure comparable results. A total of 289,022 genes were annotated across all 28 genomes. *P. rainierensis* (Col. Bob) had the highest number of genes annotated at 14,480, and *L. normandinoides* had the lowest number of genes annotated at 7,927.

RepeatModeler2 recovered TEs in all genomes in the analysis (Table 3). The mean percent of the genome occupied by TEs was generally higher in rare species than in common ones for DTs, RTs, and total TEs, which remained consistent for the number of TEs identified and the total length of TEs identified in base pairs (Fig. 3a and b; Table 3). The percent of the genome occupied by TEs ranged from 4.6% in *Bacidia gigantensis* to 69.55% in *P. rainierensis*, averaging 23.48% for all species. For DTs, the percent of genome occupied ranged from 0% in *B. gigantensis* and *Cladonia rangiferina* to 19.88% in *Sulcaria isidiifera*, averaging 4.38% among all species. RTs ranged from 3.9% in *S. deyana* to 65.49% in *P. rainierensis*, averaging 18.86% across all species.

**Table 3.** Summary of transposable element (TE) results for percent of genome occupied, TE counts, and bp length by rarity.

Species	Rarity	Total TE length (bp)			Percent of genome occupied			Counts		
		Total TE content	Total RT content	Total DT content	Total TE content	Total RT content	Total DT content	Total TE content	Total RT content	Total DT content
*Usnea strigosa*	C	8,212,277	3,441,114	4,728,657	19.04	7.98	10.96	10,228	2,743	7,244
*Cladonia rangiferina*	C	5,562,552	5,562,552	0	14.45	14.46	0	8,332	8,332	0
*Lepraria finkii*	C	9,137,759	7,650,371	1,478,615	22.37	18.71	3.62	12,985	11,628	1,342
*Lepraria neglecta*	C	7,725,769	7,002,305	723,464	18.38	16.65	1.73	10,110	9,433	677
*Lepraria normandinoides*	C	5,460,648	5,180,564	280,084	14.5	13.76	0.75	11,858	11,346	512
*Lepraria oxybapha*	C	11,123,687	9,973,157	1,150,530	25.65	22.99	2.66	13,662	12,772	890
*Bacidia rubella*	C	2,958,150	2,951,483	6,667	8.83	8.81	0.02	3,120	3,045	75
*Acarospora socialis*	C	4,806,406	4,550,964	255,442	15.43	14.61	0.82	5,113	4,928	185
*Umbilicaria muehlenbergii*	C	8,714,503	8,292,659	342,199	25.03	23.82	0.97	9,798	9,339	406
*Ramalina farinacea*	C	4,418,760	4,173,172	140,300	13.5	12.74	0.42	4,532	4,359	120
*Sticta beauvoisii*	C	9,970,174	6,161,535	3,808,639	21.36	13.2	8.15	27,429	7,374	20,055
*Pseudocyphellaria citrina*	C	11,561,917	6,077,154	5,423,121	22.4	11.78	10.49	30,395	5,862	24,407
*Punctelia rudecta*	C	6,619,243	6,599,628	19,615	15.48	15.43	0.05	6,567	6,549	18
*Letharia columbiana*	C	17,021,104	7,720,013	9,301,091	32.57	14.77	17.8	25,553	5,753	19,800
*Diploschistes muscorum*	C	23,653,310	19,923,834	3,728,012	45.89	38.65	7.23	24,618	21,275	3,329
*Letharia lupina*	C	15,398,938	15,254,465	144,473	31.27	30.98	0.29	26,357	26,043	314
	Average:	9,521,574.813	7,532,185.625	1,970,681.813	21.634375	17.45875	4.1225	14,416.0625	9,423.8125	4,960.875
*Usnea subfusca*	R	11,958,399	7,226,579	4,691,693	24.36	14.72	9.56	12,355	4,942	7,364
*Cladonia imbricarica*	R	4,945,075	4,945,075	0	13.49	13.49	0	5,048	5,048	0
*Cladonia perforata*	R	3,763,472	3,308,372	455,100	11.2	9.85	1.35	5,670	5,377	293
*Lepraria lanata*	R	15,737,969	14,439,754	1,298,215	33.41	30.65	2.77	10,668	9,685	983
*Punctelia appalachensis*	R	10,423,714	7,618,439	2,805,275	22.81	16.67	6.14	10,130	4,526	5,604
*Niebla homalea*	R	19,030,042	16,901,077	300,710	37.63	33.42	0.59	18,761	17,787	179
*Bacidia gigantensis*	R	1,523,955	1,523,955	0	4.6	4.6	0	1,850	1,850	0
*Sulcaria isidiifera*	R	35,694,133	21,237,288	14,188,738	50	29.75	19.88	39,727	12,323	27,347
*Sticta deyana*	R	5,300,682	1,700,314	3,551,567	12.18	3.9	8.18	23,012	2,844	19,989
*Pseudocyphellaria rainierensis*	R	87,343,961	82,258,960	4,658,747	69.55	65.49	3.7	131,257	123,256	7,593
	Average:	19,572,140.2	16,115,981.3	3,195,004.5	27.923	22.254	5.217	25,847.8	18,763.8	6,935.2

### Phylogenetic analyses and trait data

**Fig. 2.**
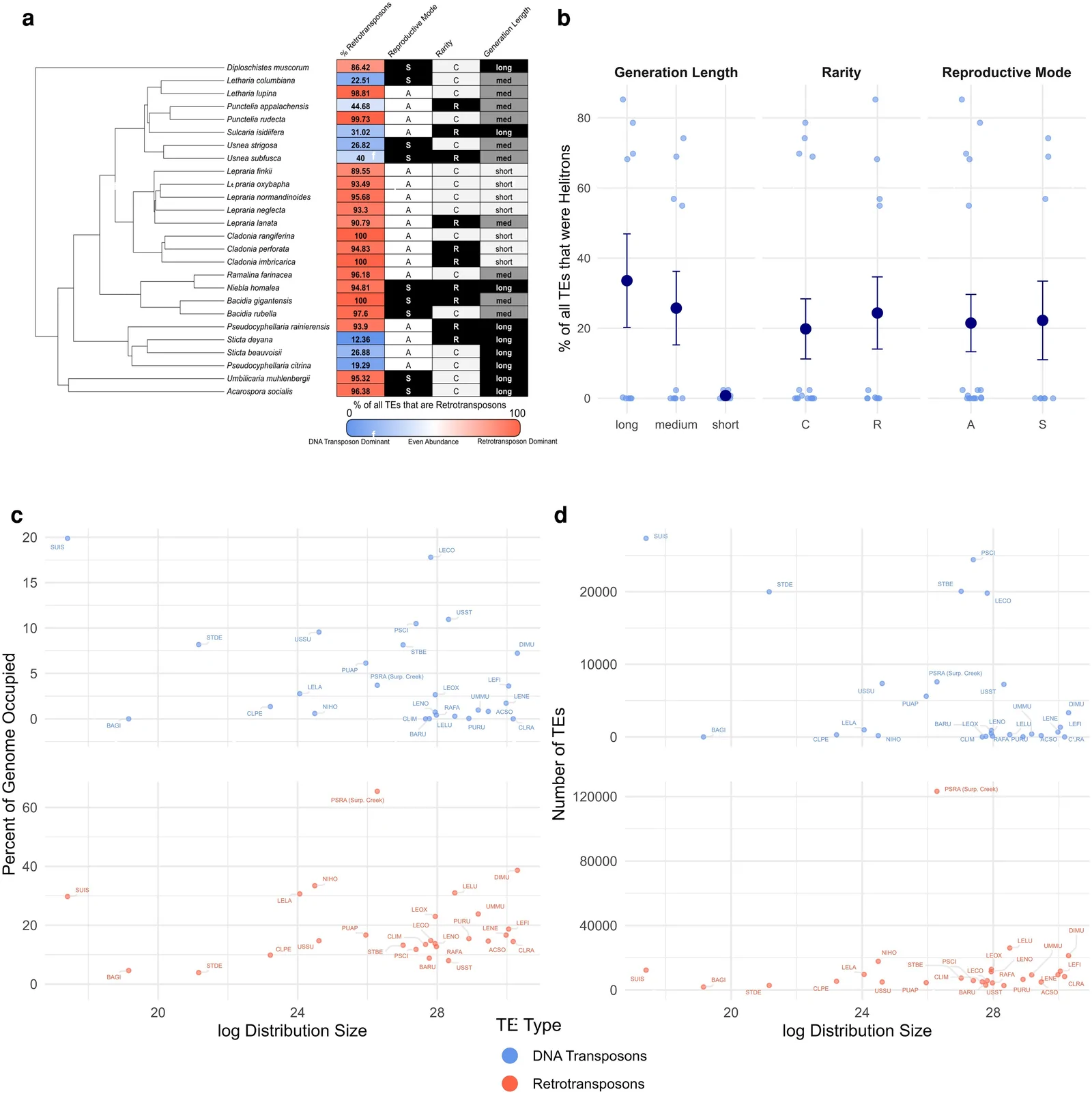
Relationship between transposable element abundance and species traits. a) Species tree of all long-read genomes in the mobile genetic element (TE) dataset, with RT abundance continuously mapped over the tree. The grid to the right of the phylogeny contains the data for the percent of total TEs that were retrotransposons (RTs), reproductive mode (sexual [S] or asexual [A]), rarity (rare [R] or common [C]), and generation length for each species. The percent of total TEs that were RTs is represented by a heat map, where boxes in red show RT dominant genomes, boxes in blue show DNA transposon (DT) dominant genomes, and white boxes show an even abundance of both. b) Dot plot representing the mean percent of all TEs that were assigned as helitrons and standard error for each group by generation length, rarity, and reproductive mode. c) Scatter plot of TE abundance values for each species, with percent of the genome occupied on the *y* axis and log of distribution size on the *x* axis. Blue dots in the top half of the figure represent DT abundance, and red dots on the bottom half represent RT abundance. d) The same scatterplot, this time representing the number of TEs in each genome on the *y* axis.

To generate a robust phylogeny for downstream phylogenetically-informed statistical analyses, we used whole genome protein sequence annotations from our focal 26 species along with a suite of publicly available fungal genomes generated with short-read data (Supplementary Fig. 2). Orthofinder uncovered a total of 593,001 genes among the 58 genomes used to generate the species tree, of which 570,340 (96%) were within the 25,506 orthogroups recovered. Three hundred ninety-six of the orthologs were present in all species in the analysis. All branch support values on the resulting species tree were 100%, except for the placement of *Cyanodermella asteris*. All nodes in the final pruned tree had support values of 100% (Fig. 2a).

Trait data were assembled from various sources for all species (Fig. 2a; Table 2). Distribution sizes ranged from 3.62e7 to 1.44e13 m^2^, with rare species averaging a distribution of 1.71e11 (SE = 3.41e10) m^2^ and common ones 5.25e12 (SE = 1.24e12; Table 2). The dataset contained 9 sexually reproducing species and 17 asexually reproducing species. There were 10 rare species and 16 common species. Nine of the species had a long generation length, 10 had a medium generation length, and 7 had a short generation length.

### Genomic TE abundance is associated with traits of rarity in lichenized fungi

We recovered a significant relationship between the percent of the genome occupied by DTs and log distribution size (PGLMM Binomial, *P*-value < 0.004; Fig. 2d; Table 4), but did not find a significant relationship between RTs and log distribution size (Fig. 2c) or total TEs and log distribution size (Table 4). The percentage of the genome occupied by total TEs was significantly higher in species with long generation lengths when compared to species with medium generation lengths (PGLMM Binomial, *P*-value < 0.004; Table 4). Further, species with long generation lengths had a significantly higher percentage of their genome occupied by DTs than those with medium generation lengths (PGLMM Binomial, *P*-value < 0.004; Table 4). The number of total TEs and DTs in the genomes of species with long generation lengths was significantly higher than in those with medium or short generation lengths (PGLMM Poisson, *P*-value < 0.004; Table 5). For both rarity and reproductive mode, no significant relationship was recovered between either trait and the percent of the genome occupied by TEs or the number of TEs in the genome (Tables 4 and 5).

**Table 4.** Binomial phylogenetic generalized linear mixed model (PGLMM) results and test values.

	Percent of genome occupied by transposable elements (TEs) (binomial PGLMM)											
	Total TEs				Retrotransposons				DNA transposons			
Factor	Value	Standard error	*Z* score	*P*	Value	Standard error	*Z* score	*P*	Value	Standard error	*Z* score	*P*
Log distribution size (m^2^)	−0.080018	0.032449	−2.466	*0.01366*	−0.016738	0.035113	−0.4767	0.6336	−0.214167	0.065526	−3.2684	**0**.**001081**
Rarity (rare:common)	0.19945	0.23864	0.8358	0.40327	0.13085	0.24007	0.5451	0.58571	0.18131	0.57579	0.3149	0.7528
Reproductive mode (sexual:asexual)	0.048272	0.258645	0.1866	0.85195	−0.090166	0.25651	−0.3515	0.7252	0.38988	0.61668	0.6322	0.52724
Generation length (short, medium, long)												
Short:long	−0.5031	0.28109	−1.7898	0.07348	−0.12523	0.31183	−0.4016	0.68797	−2.11132	0.76362	−2.7649	*0*.*005694*
Med:long	−0.82843	0.28112	−2.9469	**0**.**00321**	−0.39146	0.30531	−1.2822	0.19978	−2.09643	0.72822	−2.8789	**3.99E−03**

Bolded values are significant with a *P*-value threshold corrected for multiple testing (Bonferroni, *P*-value < 0.004). Italicized *P*-values are less than 0.05.

**Table 5.** Poisson phylogenetic generalized linear mixed model (PGLMM) results and test values.

	Number of transposable elements (TEs) in genome (Poisson PGLMM)											
	Total TEs				Retrotransposons				DNA transposons			
Factor	Value	Standard error	*Z* score	*P*	Value	Standard error	*Z* score	*P*	Value	Standard error	*Z* score	*P*
Log distribution size (m^2^)	−0.137631	0.056315	−2.444	*0.01453*	−0.010094	0.042257	−0.2389	0.8112	−0.4695	0.20868	−2.2499	*0.02446*
Rarity (rare:common)	0.1922	0.4139	0.4644	0.6424	−0.037963	0.279284	−0.1359	0.8919	−0.215	1.5342	−0.1401	0.8886
Reproductive mode (sexual:asexual)	−0.34049	0.44745	−0.7609	0.4467	−0.39944	0.29352	−1.3609	0.1736	0.90026	1.66082	0.5421	0.5878
Generation length (short, medium, long)												
Short:long	−1.0361	0.31462	−3.2931	**0**.**0009907**	−0.22728	0.35692	−0.6368	0.52427	−5.0386	1.378	−3.6564	**0**.**0002558**
Med:long	−1.60816	0.23597	−6.8151	**9.42E−12**	−0.45882	0.26773	−1.7137	0.08658	−4.5978	1.0069	−4.5662	**4.97E−06**

Bolded values are significant with a *P*-value threshold corrected for multiple testing (Bonferroni, *P*-value < 0.004). Italicized *P*-values are less than 0.05.

We assessed if either RTs or DTs were enriched in rare species by calculating the proportion of RTs relative to all TEs in each genome, wherein all TEs that were not classified as RTs were classified as DTs. We found a significant positive association between log distribution size and percent of all TEs identified as RTs, as distribution size increases, RTs increase and, conversely, DTs decrease. Thus, a greater proportion of TEs are DTs in species with smaller distributions. Additionally, we found that species with long generation lengths were significantly enriched with DTs when compared to species with medium and short generation lengths, which had more RTs (PGLMM Binomial, *P*-value < 0.0125; Fig. 2b; Table 6). Because the largest number of DTs in most species were attributed to the category of Rolling Circles (i.e. Helitrons and Unknown RCs; Supplementary Tables 3 to 5), we also tested if the relative abundance of Rolling Circles vs all other DTs was driving these relationships, and found again that Helitrons were enriched in species with small distribution sizes and long generation lengths (Table 6; Fig. 2b).

**Table 6.** Transposable element (TE) enrichment binomial phylogenetic generalized linear mixed model results and test values.

	Transposable element (TE) enrichment (number of retrotransposons or helitrons/total number of TEs)							
	Retrotransposons				Helitrons			
Factor	Value	Standard error	*Z* score	*P*	Value	Standard error	*Z* score	*P*
Log distribution size (m^2^)	0.41182	0.15063	2.7341	**0.006255**	−0.47254	0.17555	−2.6918	**0**.**007107**
Rarity (rare:common)	−0.42286	1.16509	−0.3629	0.7166	0.49875	1.4637	0.3407	0.7333
Reproductive mode (sexual:asexual)	−0.75556	1.27935	−0.5906	0.5548	−0.45197	1.56495	−0.2888	0.7727
Generation length (short, medium, long)								
Short:long	3.78965	0.88408	4.2865	**1.82E−05**	−4.4195	1.5166	−2.914	**0**.**003568**
Med:long	3.41515	1.14582	2.9805	**0**.**002877**	−2.8219	1.588	−1.777	0.075567

Bolded values are significant with a *P*-value threshold corrected for multiple testing (Bonferroni, *P*-value < 0.0125). Italicized *P*-values are less than 0.05.

### Genome architecture analysis

No statistically significant relationships were detected between TE proximity to genes and trait data after applying a Bonferroni correction (adjusted *P*-value threshold = 0.00625; Gaussian PGLMM; Supplementary Table 6). However, possible trends were observed for longer generation length and asexual reproduction (Fig. 1b and c, respectively; Supplementary Fig. 3), which were associated with decreased TE distance to genes (*P* < 0.05), particularly for RTs and DTs in models without transcriptional directionality. The 2 *P. rainierensis* individuals collected from different populations showed high sequence similarity, including a similar number of TEs (Fig. 1b; Supplementary Tables 3 and 4), and a pairwise alignment of the 2 genomes only revealed one small inversion on contig 4 (Fig. 1d).

## Discussion

### Genome metrics and architecture

We generated 18 new long-read genomes for Lecanoromycetes lichenized fungi, contributing substantially to the availability of genomic resources for a group of organisms that is underrepresented in scientific research. Most genome metrics were in line with previously published long-read lichenized fungal genomes, with BUSCO scores greater than 90% and the size of most genomes ranging from 30 to 50 Mb (Table 1). We noticed that *P. rainierensis* (131 Mb) and *S. isidiifera* (71 Mb) had much larger genomes than the rest. Upon annotating these genomes, it became clear that this size difference was due to high TE content, as *P. rainierensis* and *S. isidiifera* had as much as 69 and 50% of their genomes occupied by TEs, respectively, while most of the other genomes ranged from 10% to 30%. Sequencing a second *P. rainierensis* individual confirmed that the unusually high TE, and especially RT content, was potentially a trait of the species’ genome and not specific to an individual population. *P. rainierensis* and *S. isidiifera* are both geographically restricted species, endemic to declining habitats, which led us to hypothesize that TE abundance may be connected to rarity in lichenized fungi. Assembling the repetitive regions of short-read genomes is difficult without a long-read reference (Kellogg 2015). The completion of these new genomes from long-read sequencing therefore provided us with a unique opportunity to investigate TEs and genome architecture in lichenized fungi.

### DTs, not RTs, are associated with small distribution sizes

**Fig. 3.**
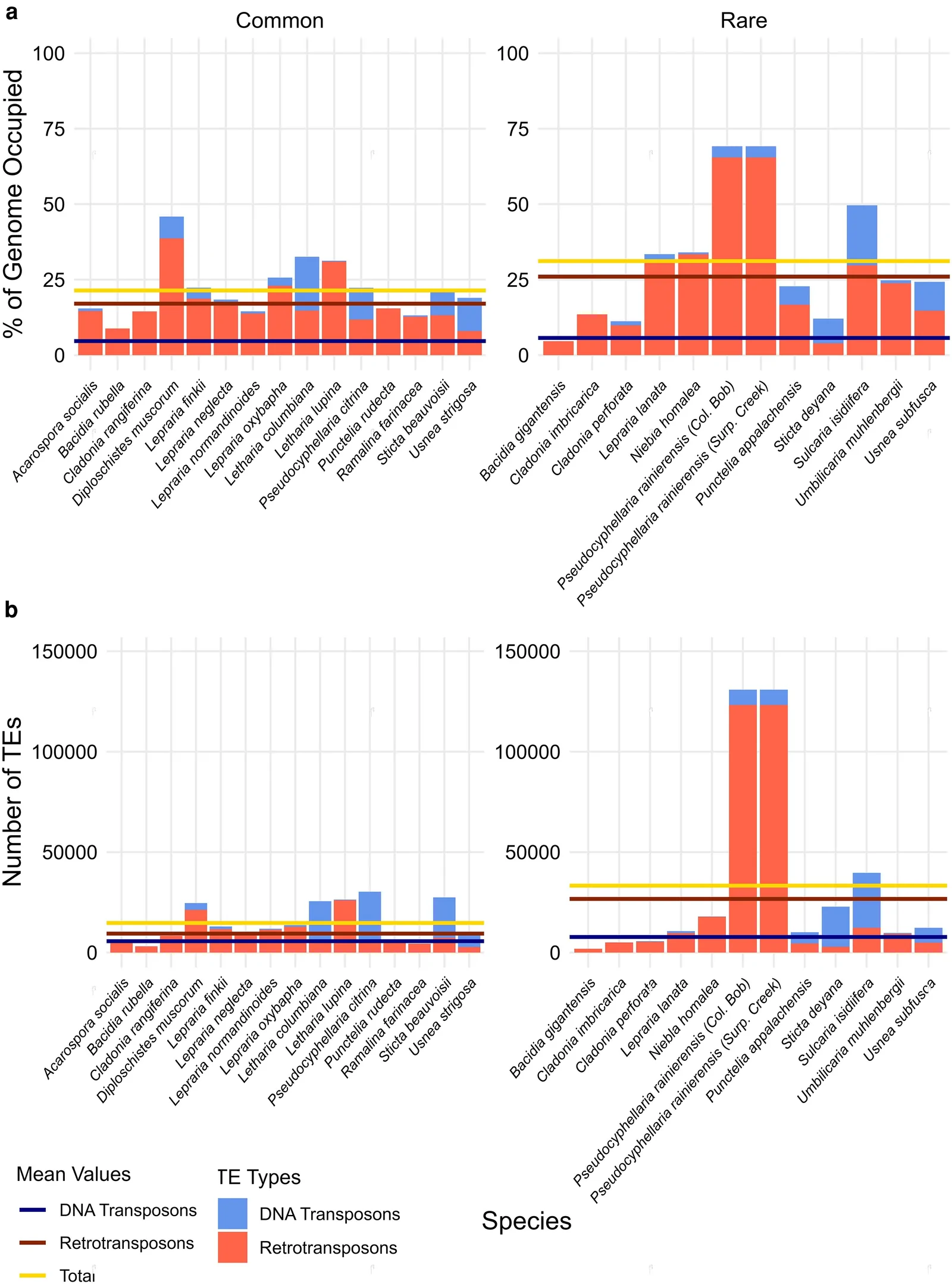
Overall genomic TE results. a) Percent of the whole genome occupied by transposable elements (TEs) per species, with retrotransposons represented in red (bottom bar) and DNA transposons represented in blue (top bar). The left grid represents all species assigned as common in the dataset, with the right grid containing the rare species. Horizontal lines indicate the mean value for rare or common species for each TE type: DNA transposons (dark blue), retrotransposons (dark red), and total TE content (yellow). b) Number of TEs in each species genome.

We hypothesized that RTs would have a strong association with rarity because they have been linked to environmental stress and disturbances, by which rare organisms are often disproportionately impacted (Cavrak et al. 2014; Miousse et al. 2015; Milyaeva et al. 2023). For example, Esnault et al. (2019) found that exposing *Schizosaccaromyces pombe* to CoCl2 increased the transposition of individual Tf1 elements 6- to 139-fold, and that specific insertion modalities that increased metal resistance reproducibly appeared and proliferated across generations, linking these transpositions with direct increases in fitness. Linscott et al. (2022) analyzed the genome of a calcareous substrate endemic land snail, *Oreohelix idahoensis*, and found a high abundance of long terminal repeat (LTR) RTs relative to other land snail genomes, with evidence suggesting recent expansions in LTR families. However, they did not identify any patterns related to DTs. A study of the endangered fern *Vandenboschia speciosa* by Ruiz-Ruano et al. (2021) also recovered a large quantity of LTR RTs in the species' genome, constituting 51% of the genome as opposed to the 5.6% of the genome occupied by DTs. Similarly, RTs were dominant in most of the genomes in our analysis when compared to DTs (Fig. 3a and b), a phenomenon consistent with data from other fungal genomes (Castanera et al. 2017). After evaluating our dataset for phylogenetic signal, we used phylogenetic generalized linear mixed models to test for correlations between TE content and life history traits in lichenized fungi while accounting for expected genetic similarities due to shared ancestry. Our statistical results showed that RTs were not the primary driving force of the patterns observed in our data. Instead, we found a significant negative association between TEs and log distribution size, such that species with smaller distribution sizes had a higher percentage of their genome occupied by TEs (Table 4). While small distribution sizes are commonly associated with rarity, they may also be correlated with small population sizes and greater influence of stochastic events on distributions and populations, especially in rare species (Thompson et al. 2002; Murray et al. 2017). Our results could suggest that, overall, rarity is less important as a contributing factor to TE abundance than underlying demographic processes associated with small distribution sizes. The percent of the genome occupied by TEs was one of the datasets in which we did not identify significant phylogenetic signal (Supplementary Table 1). While this could support our conclusion by affirming that phylogenetic relatedness alone does not explain our results, it is important to note that phylogenetic signal can be difficult to detect in small datasets (fewer than 16 taxa) with closely related individuals and little trait variance (Ives and Garland 2014). Our dataset included 26 taxa and substantial trait variance, but the overrepresentation of the genus *Lepraria* in our dataset may reduce the strength of results for analyses where a strong phylogenetic signal was not detected.

### Helitrons are significantly enriched in lichenized fungi with small distribution sizes

We found that the genomes of species with smaller distribution sizes and those with longer generation lengths were significantly enriched in DTs, specifically, Helitrons and unknown Rolling Circles (Fig. 2b, Table 6). Rolling Circles are one of the few DTs whose mechanism of movement enables them to self-replicate (Barro-Trastoy and Köhler 2024). Thus, while our hypothesis that RTs would be significantly enriched in rare species was not supported, our findings may still be consistent with our predicted mechanism of unchecked self-replication of TEs in stressful environmental conditions. Helitrons have been previously identified in the genome of *Umbilicaria pustulata*, where they comprise the majority of the Class II elements identified (Dal Grande et al. 2021). Notably, approximately 10% of the Helitrons found by Dal Grande et al. (2021) were highly differentiated insertions between populations from different ecotypes, potentially associating them with environmental stress. We expected to recover intraspecific variation in the high TE content of *P. rainierensis*, but did not observe a difference in the abundance of TEs of any type in the genomes of 2 individuals sequenced from different populations (Supplementary Tables 3 to 5), nor did we observe any impacts on the structure of the genome which could be caused by active TEs (Fig. 1d). Given that *P. rainierensis* grows in a narrow range of ecological conditions and in old growth habitat that is presumably very stable, it may not be a good model for understanding intraspecific TE variation triggered by environmental influence, like what was observed in Dal Grande et al. (2021).

Helitrons are unique among DTs in that they have the ability to “capture” and express host genes and regulatory elements (Barbaglia et al. 2011). This interaction poses a conflict for host TE defenses, as silencing of Helitrons results in the silencing of captured host genes. A study by Muyle et al. (2020) analyzed the genome of maize to identify Helitrons with captured host genes and their regulatory markings. They found that, on average, Helitrons with captured host genes were less impacted by host silencing when compared to other Helitrons. Additionally, Helitron-captured host genes were more impacted by host silencing when compared to other host genes not captured by a Helitron (Muyle et al. 2020). It is important to note that fungi have different mechanisms to prevent TE transposition than plants (e.g. RIP), which could impact the relevance of these results to our study system. In the oyster mushroom *Pleurotus ostreatus*, Helitrons were found to have captured fragments of fungal genes and retroviral DNA originating from plant and animal hosts, and transcriptional data confirmed that these fragments were expressed to varying degrees (Castanera et al. 2014). Helitrons have variable transcriptional activity among species of Fungi, and Castanera et al. (2014) suggest that they may be less transcriptionally active in Ascomycota than Basidiomycota. All taxa investigated here are Ascomycota, suggesting that general patterns in Helitron activity may vary within phyla, but transcriptional data are required to test this hypothesis. *Starships* are large, autonomous TEs identified in many fungal lineages (Gluck-Thaler and Vogan 2024) and recently in lichenized fungi (Tagirdzhanova et al. 2025). Like helitrons, *Starships* can cause genomic restructuring, and they contain accessory genes that are then expressed by the host (Gluck-Thaler et al. 2025; Sato et al 2025). While we did not investigate the presence of *Starships* in our sequenced genomes, this data could provide further insight into the genomic plasticity that TEs can generate in response to life-history traits. The interactions among the modification of gene expression through the activity of Helitron transposition and silencing, host-gene cargo, and rarity remain to be investigated, and, similarly, the role of *Starships* in a similar context.

### Generation length impacts TE abundance

Generation length is a complex trait related to both life span and reproduction. In lichens, generation length is primarily driven by lifespan with a minor influence of reproductive mode (Yahr et al. 2024). Both sexually and asexually reproducing species may exhibit shorter or longer generation lengths, and, indeed, reproductive mode is not correlated with generation length in our dataset (Supplementary Table 2). Sexual reproduction may be predicted to either facilitate the spread of TEs to new populations or reduce TE abundance by increasing purifying selection (Bestor 1999; Arkhipova and Meselson 2004). Testing these competing hypotheses, Nowell et al. (2021) found that in Bdelloid rotifers, which reproduce exclusively asexually, DTs evolved in a primarily random fashion, with no apparent relationships between reproductive mode and TE content being recovered. We similarly did not find any significant relationships between TE abundance and reproductive mode, but a marginal trend (*P* < 0.05) was uncovered for TE spatial distribution, indicating that asexually reproducing species may experience higher instances of TE and gene colocalization (Supplementary Table 6). In termites, TE transcription profiles are found to cluster based on both age group and reproductive caste (Post et al. 2022). Previous studies have found surprisingly low rates of somatic mutation and high genome integrity in plants and fungi with long life spans (Schmid-Siegert et al. 2017; Anderson et al. 2018; Hofmeister et al. 2020). In our study, species with long generation lengths had a higher number of TEs, specifically DTs, in their genomes than those with short or medium generation lengths, contradicting these previous findings. Symbiont interactions are known to have impacts on genome architecture in symbiotic organisms (Looney et al. 2021; Li et al. 2025). Transitions between symbiotic states in ectomycorrhizal fungi, for example, result in gains and losses of genes related to secondary metabolism and enzymatic activity, and expansion and aggregation of TEs in the genome (Looney et al 2021). However, the potential relationship between symbiont type and TE content in lichens remains to be studied, as we did not have sufficient representation of different symbiotic states in our dataset to investigate this question. The potential interactions among life span, reproductive mode, generation length, and TE dynamics in the context of complex lichen life cycles warrant further investigation.

### Ploidy, life cycles, and TEs

Lichenized fungi are commonly assumed to consist of haploid mycelia, which only enter a brief dikaryotic diploid phase upon the formation of ascomata during sexual reproduction (Tripp and Lendemer 2018). Thus, species with long generation lengths, or those that form only haploid asexual spores and propagules, spend the vast majority of their time in a haploid state. Hiltunen et al. (2022) suggest that higher TE activity is associated with haploidy. They compared the expression level of TEs in different phases of the life cycle of the fairy-ring mushroom, *Marasmius oreades*, and found that DT mutagenesis was most active during the haploid phase of the life cycle, and the formation of dikaryotic hyphae after sex and fusion resulted in a highly stable genome with little DT activity. If TE activity is associated with a haploid genome state (Hiltunen et al. 2022), haploid species may be the most at risk for TE accumulation and resultant genome mutations. As the available genomic resources for lichenized fungi increase, it has been revealed that the assumption that lichen's vegetative hyphae are always formed by haploid mycelia is not true, which may be an additional factor contributing to TE variation (Tripp et al. 2017; McKenzie et al. 2020). In our study, all of the genomes originated from haploid thalli except that of *Letharia lupina*, which is triploid, and *Letharia columbiana*, which is diploid (McKenzie et al. 2020); thus, this hypothesis remains to be tested.

Bryophytes are another group of organisms with a haploid-dominant life cycle. They also share a poikilohydric physiology with lichens. The TE landscape in bryophyte genomes is not well characterized, but it is known that variations in genome size among species are not fully explained by whole-genome duplication events (Szövényi et al. 2021). A study by Patel et al. (2025) found that hornworts had the smallest genomes with the least size variation when compared to liverworts and mosses. Variability of TE abundance has been observed in several groups of nonvascular land plants, and TE activity could be one possible explanation for size variation among this group (Lang et al. 2018; Linde et al. 2023; Kirbis et al. 2025). Due to the alternation of sexual and asexual reproductive modes in the bryophyte life cycle, increased focus on generating genomic resources for bryophyte TEs may provide further insights into the intersecting relationship between generation length, reproductive mode, and TE activity in haploid-dominant organisms using an evolutionarily independent comparison point.

## Conclusion

Previous studies of single rare species have recovered large abundances of RTs occupying their genomes, resulting in a focus on RTs and their association with rarity and genomic stress in conservation research (Abascal et al. 2016; Ruiz-Ruano et al. 2021; Linscott et al. 2022). We similarly found 2 rare species with extraordinarily high RT content in their genomes. However, by investigating TE abundance in a multispecies comparative phylogenetic framework, we uncovered unexpected associations between distribution size, generation length, and DT abundance in lichenized fungi. Specifically, DTs, and especially self-replicating Helitrons, were significantly enriched in species with small distribution sizes and long generation lengths, while RTs were not. Further research is needed to determine if these patterns are present in other groups of organisms, which will require continued focus on the development of high-quality, long-read reference genomes for more species. Further, additional genomic data and greater sampling sizes may change the patterns recovered here, which emphasizes the importance of continuing to generate reference genomes for a wide variety of species, such as the ones published in this study. Assessing rarity for effective species conservation requires a multifaceted approach. TEs, particularly DTs, may serve as a genomic signature of rarity, providing additional context for conservation management and strengthening our understanding of the processes that increase extinction risk.

## Supplementary Material

jkag153_Supplementary_Data

## Data Availability

Sequence data are deposited at the National Center for Biotechnology Information (BioProject PRJNA1270085). Assemblies and annotations are available on FigShare (https://doi.org/10.6084/m9.figshare.32248011). Supplemental material available at G3 online.
