# Supplementary material for: Helitrons are enriched in lichenized fungi with long generation lengths and small distribution sizes
Source: G3 (Bethesda). 2026 Jun 16;16(9):jkag153. doi: 10.1093/g3journal/jkag153 (PMC13535494; doi:10.1093/g3journal/jkag153)
Supplement: Supplementary file 1 [file jkag153_supplementary_data.zip › Supplemental_Material_Legends_G3-2026-406759.docx]

**Supplementary Figure and Table Legends**

**Supplementary Figure 1.** Alignment of *Sticta deyana* and *Sticta beauvoisii* genomes. Gridlines delineate contigs. Total genome length is represented on the bottom and left axes.

**Supplementary Figure 2.** Unpruned species tree produced with Orthofinder v2.5.5 using annotated protein-coding genes from whole genome sequences All nodes, except *Cyanodermella asteris*, have support values of 100%.

**Supplementary Figure 3.** Double faceted heatmap of the number of 100kb windows in each genome (represented by the gradient of low values (light purple) to high values (dark purple)) with a specific number of genes (x-axis) and transposable elements (TEs, y-axis). Each position in the grid shows the distribution of TEs and genes in all 100kb adjacent windows from genomes belonging to species with the combination of the corresponding reproductive mode and generation length category. ‘All’ category represents all windows belonging to species with the trait in the row below or column to the left.

**Supplementary Table 1. Tests for Phylogenetic Signal in TE (Transposable Element) Data.** Significant p-values less than 0.05 are bolded.

**Supplementary Table 2. Statistical tests to Evaluate Correlations Between Trait Data.** Significant p-values less than 0.05 are bolded.

**Supplementary Table 3. Percent of the Genome Occupied by TEs (Transposable Elements) in each Genome by Type of TE.**

**Supplementary Table 4. Count of TEs (Transposable Elements) in each Genome by Type of TE.**

**Supplementary Table 5. Total Length (base pairs) of TEs (Transposable Elements) in each Genome by Type of TE.**

**Supplementary Table 6. Spatial Distribution of TEs within Genomes Gaussian Phylogenetic Generalized Linear Mixed Model Results and Test Values.** No p-values were significant with a corrected significance threshold of 0.00625 (Bonferroni correction). P-values less than 0.05 are italicized.
